# Supplementary material for: Evaluating Experiences in a Digital Nutrition Education Program for People With Multiple Sclerosis: A Qualitative Study
Source: Health Expect. 2024 Aug 29;27(5):e70012. doi: 10.1111/hex.70012 (PMC11361266; doi:10.1111/hex.70012)
Supplement: Supplementary file 1 — Supporting information. [file HEX-27-e70012-s002.docx]

Appendix A. Consolidated criteria for reporting qualitative studies (COREQ): 32-item checklist [[17](#_ENREF_17)].

DOMAIN 1: Research team and reflexivity

| Items | Guide question/description | Reported on Page # |
| --- | --- | --- |
| *Personal characteristics* | | |
| Interviewer/facilitator | Which author/s conducted the interview or focus group? | 3  3  3 |
| Occupation | What was their occupation at the time of the study? |  |
| Gender | Was the researcher male or female? |  |
| *Relationship with participants* | | |
| Interviewer characteristics | What characteristics were reported about the Interviewer/facilitator? e.g. *Bias, assumptions, reasons and interests in the research topic* | 3 |

DOMAIN 2: Study design

| Items | Guide question/description | Reported on Page # |
| --- | --- | --- |
| *Theoretical framework* | What methodological orientation was stated to underpin the study? e.g. grounded theory, discourse analysis, ethnography, phenomenology, content analysis | 3 |
| *Participant selection* | | |
| Sampling | How were participants selected? | 2 |
| Sample size | How many participants were in the study? | 3 |
| Method of approach | How were participants approached? e*.g. face-to-face, telephone, mail, email* | 2-3 |
| Non-participation | How many people refused to participate or dropped out? Reasons? | 2 |
| *Setting* | | |
| Description of sample | What are the important characteristics of the sample? e.g. demographic data, date | 3-4 |
| *Data collection* | | |
| Interview guide | Were questions, prompts, guides provided by the authors? Was it pilot tested? | 3 and Appendix B |
| Audio/visual recording | Did the research use audio or visual recording to collect the data? | 3 |
| Duration | What was the duration of the interviews or focus group? | 4 |
| Data saturation | Was data saturation discussed? | 3 |
| Transcripts returned | Were transcripts returned to participants for comment and/or correction? | 3 |

DOMAIN 3: Analysis and findings

| Items | Guide question/description | Reported on Page # |
| --- | --- | --- |
| *Data analysis* | | |
| Number of data coders | How many data coders coded the data? | 3 |
| Derivation of themes | Were themes identified in advance or derived from the data? | 3 |
| Software | What software, if applicable, was used to manage the data? | 3 |
| *Reporting* | | |
| Quotations presented | Were participant quotations presented to illustrate the themes/findings? Was each quotation identified? e.g. participant number | 5-9 |
| Clarity of major themes | Were major themes clearly presented in the findings? | 5-9  5-9 |
| Clarity of minor themes | Were minor themes clearly presented in the findings? |  |
