# Supplementary material for: Evaluating Experiences in a Digital Nutrition Education Program for People With Multiple Sclerosis: A Qualitative Study
Source: Health Expect. 2024 Aug 29;27(5):e70012. doi: 10.1111/hex.70012 (PMC11361266; doi:10.1111/hex.70012)
Supplement: Supplementary file 2 — Supporting information. [file HEX-27-e70012-s001.docx]

Appendix B. Semi-structured interview guide.

Hi [participant], my name is [researcher].

I am from the Eating Well with MS nutrition program that you were enrolled in recently. Thank you for taking the time to chat with me. I would like to ask you some questions over the next 30-45 minutes about your involvement in the program.

We’d like you to help us understand what parts of the program you found helpful or not helpful. Your views are really important to help to improve this program. Your experiences are helpful for us to make this program better, so please feel free to give us your honest opinion.

**A few important points to remember are:**

- There are no right and wrong answers – we’re interested in what you think
- You don’t have to answer any questions that make you feel uncomfortable or that you’d prefer not to answer – please just let me know that you’d like to pass
- If anything that I say isn’t clear or you have more you’d like to add - please stop me at any time

**Before we start, would you mind if I record your answers so that we don't miss any details? (Start phone recording with permission).**

*Motivation*

- Could you tell me why you signed up for the nutrition program?
- Was there anything you were hoping to get out of the program?

**You completed [all of the modules / the first two / the first three modules / Module 1] of the program. Could you tell me if this was as much of the program as you expected to get through in the 7 weeks?**

- [non-completers]: can you tell me about some of the reasons why you didn’t get to all of the modules? [same for people who completed Module 1]
- If you did the program again, what sorts of things do you think would help you to get through all of the modules? [same for people who completed Module 1]

**I’m now going to ask about any changes you made to your eating during the program.**

Have you made any changes to your eating habits as a result of taking part in the program?

Can you think of anything that helped you to make these changes? (prompt: planning ahead, recipes, knowledge about food groups and nutrients)

**I’d like to ask some questions about your eating habits and your cues to eating.**

As a result of being in the program have you formed any new food habits that are becoming routine or automatic for you? *(prompt: cues to eating, type of foods selected, where or when you eat)*

- - Can you tell me more about this? (*What* *helped* *you develop the habits*)?

*Social support*

Thinking about the people around you, such as friends, family, and colleagues. Can you tell me if anyone influenced your efforts around changing your eating habits during the program?

As a result of being in the program, do you think you may have influenced anyone else around you to make changes to their eating habits?

Can you think of anything else that we *could* have provided that might have helped you achieve your goals that related to your eating habits?

*Knowledge*

As a result of the program, did you learn anything new about how the food recommendations for people with for MS?

Did you learn anything new about your own eating habits and how that compares to what’s recommended?

*Opportunity*

**Did you complete the online Healthy Eating Quiz?**

[If yes] The Healthy Eating Quiz website gave you some feedback on your eating habits, along with some suggestions on how to improve your scores for the food groups and overall

- Did you find this quiz and the suggestions useful or not useful in relation to your eating habits and setting your goals?
- If you tried some of the tips:
  - were there any suggestions or tips that you found easier to do?
  - were there any suggestions or tips that you found more challenging?

**Finally, we’d like to know your thoughts on how you think the program could be improved for a future version.**

- Overall, have you got any ideas on how the program could be improved to help people with MS improve their eating habits?
- What did you find to be the most useful part of the program?
- What did you find to be the least useful part of the program?
- I’d like to know your thoughts on some of the specific parts of the program. Briefly, can you tell me what you thought about the:
  - Activity and resource book (prompt: did you use it / how much)
  - Videos
  - Discussion board forums (prompt: did you post or read without posting?)
  - Recipe booklets
  - Label reading card

**That's the end of my questions. Is there anything else you’d like to add that we haven’t covered?**
